# *Notes from the Field:* Hepatitis A Outbreak Associated with Drug Use and Homelessness — West Virginia, 2018

**DOI:** 10.15585/mmwr.mm6814a4

**Published:** 2019-04-12

**Authors:** Erica Wilson, Megan G. Hofmeister, Shannon McBee, Janet Briscoe, Erica Thomasson, R. Henry Olaisen, Ryan Augustine, Eliana Duncan, Sapna Bamrah Morris, Loretta Haddy

**Affiliations:** ^1^Epidemic Intelligence Service, CDC; ^2^Wisconsin Division of Public Health; ^3^Division of Viral Hepatitis, National Center for HIV/AIDS, Viral Hepatitis, STD, and TB Prevention, CDC; ^4^West Virginia Bureau of Public Health; ^5^Kanawha-Charleston Health Department, West Virginia; ^6^Division of Vital Statistics, National Center for Health Statistics, CDC; ^7^Division of Tuberculosis Elimination, National Center for HIV/AIDS, Viral Hepatitis, STD, and TB Prevention, CDC.

In March 2018, the Kanawha-Charleston Health Department (KCHD) in West Virginia began investigating a cluster of reported hepatitis A virus (HAV) infections. Twelve specimens tested by CDC’s Division of Viral Hepatitis laboratory confirmed that patients were infected with an HAV strain (genotype 1B) reported in ongoing hepatitis A outbreaks in multiple states, primarily among persons who use drugs and persons experiencing homelessness (*1*). In August 2018, because of ongoing reporting of cases, the West Virginia Bureau of Public Health requested epidemiologic assistance from CDC in responding to the outbreak.

Upon retrospective review, KCHD identified a total of 664 outbreak-associated hepatitis A cases that occurred from January 1, 2018 to August 28, 2018. Outbreak cases met the 2012 Council of State and Territorial Epidemiologists’ case definition for an acute hepatitis A infection* and had either an epidemiologic link to an identified outbreak case, a laboratory specimen matching the outbreak strain, or occurred in a person at high risk for infection (e.g., reported injection or noninjection drug use, experienced homelessness or unstable housing, or was recently incarcerated) or who resided in a county where the outbreak genotype had been previously identified through laboratory testing. Median age of the patients was 37 years (range = 14–77 years); 398 of the patients (60%) were male, 380 (57%) were hospitalized, and one (0.1%) died. Current or past illicit drug use was reported by 540 (81%) patients, and being homeless or having a transient living situation was reported by 100 (15%). Evidence of past or current hepatitis C virus infection was identified in 314 (47%) outbreak-associated cases, and 65 (10%) patients had evidence of past or current hepatitis B virus infection.

HAV is typically shed in the stool of infected persons and primarily spread by the fecal-oral route, either through direct person-to-person contact or consumption of contaminated food or water. In this outbreak, transmission was primarily person-to-person among persons with a current or past history of injection or noninjection drug use.

Hepatitis A is a vaccine-preventable disease; vaccination is the primary method for stopping an outbreak (*2*). Vaccination measures were undertaken at both the state and county level in an effort to control the outbreak. Statewide hepatitis A vaccination initiatives in August 2018 included vaccination at four regional jails, through harm reduction programs, and at a large comprehensive drug treatment center, as well as provision of vaccination toolkits to 40 federally qualified health centers. Vaccination campaigns by KCHD targeted populations at high risk for HAV infection and included opt-out vaccination upon entry into homeless shelters; vaccinations at meal centers, drop-in centers, and other locations where services are provided to persons experiencing homelessness; and, through local emergency medical services, vaccination of persons who used drugs or were close contacts of patients with confirmed HAV infection. Mapping of outbreak-associated cases and administration of hepatitis A vaccine to adults in KCHD’s catchment area were used to guide vaccination strategies. As of February 2019, the statewide outbreak was ongoing, with 74 new outbreak-associated cases reported during January; however, fewer than five of those were in Kanawha County.

In other states experiencing similar person-to-person hepatitis A outbreaks, hepatitis A vaccination campaigns have successfully targeted populations at risk by vaccinating in emergency departments and at syringe exchange programs, jails, and drug treatment facilities (*3*). Increasing vaccination coverage among groups at high risk for HAV infection as recommended by the Advisory Committee on Immunization Practices (*2*,*4*,*5*) can slow ongoing outbreaks and prevent future outbreaks. Engaging partners to provide hepatitis A vaccine to persons at highest risk at all possible points of contact with the health care system and service providers might help improve vaccination coverage among groups at high risk.

## Footnotes

* https://wwwn.cdc.gov/nndss/conditions/hepatitis-a-acute/case-definition/2012/.
